# On the Nature and Structural Characteristics of Cancer, and of Those Morbid Growths Which May Be Confounded with It

**Published:** 1840

**Authors:** 


I. ON THE NATURE AND STRUCTURAL CHARACTERISTICS OF CANCER, AND OF THOSE MORBID GROWTHS WHICH MAY BE CONFOUNDED WITH IT. By *J. Müller*, M.D. Professor of Anatomy and Physiology in the University of Berlin, &c. Translated from the German, with Notes, by *Charles West*, M.D. Graduate in Medicine of the University of Berlin. Illustrated with numerous Steel Plates and Wood Engravings. Part I. Sherwood and Co. London, 1840.

II. CYCLOPÆDIA OF PRACTICAL SURGERY, Part VI.—CANCER. By *W. H. Walshe*, M.D.

IN the last number of this Journal we took up the work whose title stands first at the head of this article, and introduced to our readers that part of it which treats of Carcinoma. We now turn to the second division of it, appropriated to some of those morbid growths which may be confounded with Carcinoma—Enchondroma—Adipose Tumors—and Compound Cystoid and Cystosarcomatous Growths.

### I. OF CARTILAGINOUS TUMORS, OR ENCHONDROMA.

M. Müller remarks, that the term Chondroid has been applied to many morbid structures which differ essentially from cartilage. He proposes to describe under the name of Enchondroma, or Chondroma—true chondroid growths possessed of the characters to be described.

### 1. *General Description of Enchondroma.*

"Enchondroma," says M. Müller, "is a fungoid growth proceeding from bones, or from soft parts, as, for instance, from glands, and curable by amputation. It forms a spheroidal, not lobulated tumor, which equals or even exceeds the size of the fist. When it appears in soft parts, it is furnished with a thin covering, resembling cellular tissue; in the bones, where its occurrence is more frequent, it retains the periosteum as its investing membrane. This disease presents itself as a soft expansion of the bone, developed either within its interior, or, more rarely, from its periphery. In the former case it is covered not only by the periosteum, but also by the bone itself, which is sometimes expanded to an extreme thinness. In some instances, indeed, this bony shell is not entire, and its only remains are a few thin isolated bony laminæ on the surface of the tumor. When the growth springs from the circumference of the bone it does not necessarily have any osseous investment. The articular surfaces of the bones are usually but very little altered by this disease, often they are not at all affected, and even in instances where the phalanx of the finger has been converted into a round tumor as large as an orange, the articular surfaces are in most cases unchanged in structure, and maintain their proper position. How great soever may be the expansion of the bone, the disease seldom advances beyond the articular surface, and if two neighbouring phalanges of a finger form this expansion, the two tumors very rarely become confounded with each other. The occurrence of anchylosis also is unusual, though it took place in a case of enchondroma described by Mery.

The parts covering the enchondroma generally remain unaltered, notwithstanding the large size to which the tumor may attain. This circumstance, together with the slow, painless development of the tumor, and its existence for ten or twenty years without producing any injurious effect on the constitution, sufficiently prove its benignant character. The contents of the tumor are soft: when it is developed in or on the bones small spiculæ of osseous matter are usually intermixed with its tissue, although their presence is not constant. The parenchyma of the growth usually displays, when divided, two different constituents, distinguishable with the naked eye; the one a fibro-membranous substance, the other grey, pellucid, and resembling cartilage or very firm jelly. The fibro-membranous part, which is but seldom absent, forms cells both large and small, some equalling or even exceeding the size of a pea; and the larger cells often containing smaller ones in their cavity. Within these cells is contained the other substance, which is grey, somewhat pellucid, differing from cartilage in being more soft, and more nearly resembling in consistence the soft hyaloid cartilage of cartilaginous fishes: in some instances, indeed, it is not firmer than very firm jelly. These masses are easily removed from the cells, and are then found to be very friable. Like the hyaloid cartilages of cartilaginous fishes, this substance preserves its pellucid character in alcohol. The intervention of membranous structures connects the transparent cartilaginous masses, and imparts to enchondroma a conglomerated appearance, which does not occur in any other form of exostosis. The slight inequalities which may be seen on the surface of an enchondromatous tumor indicate this conglomerated structure of its interior." 99.

The fibro-membranous part, proceeds our author, appears, under the microscope, to be composed of an interweaving of transparent fibres; the hyaloid mass, however, so closely resembles cartilage, as to suggest to the author the name he has applied to this form of tumor. Real cartilage, even that of the cartilaginous fishes, contains, scattered through its substance, oval or round semi-transparent cartilage corpuscules,* and cells containing granules, or smaller cellules; and precisely such microscopic corpuscules are contained in the hyaloid mass. But, how similar soever to cartilage the hyaloid mass may appear, when viewed by the naked eye, or by the aid of a microscope, yet the fibro-membranous capsules or cells which usually intersect the whole of the tumor distinguish its texture from that of true cartilage. These membranous structures contain blood-vessels.

M. Müller has seen many specimens of enchondroma both in Germany and England. The bones are the most frequent seat of it. In only four out of thirty-six cases, was it found occupying the soft parts. In all of these four cases the parts affected were glandular structures, namely, in one

* "These bodies were first observed by Professor Purkinje, in human cartilage, and were described by him in a dissertation by Deutsch, De penitiori ossium structura observationes, *Vratisl,* 1834."

instance the parotid gland, in another the mammary gland, and in the remaining two the testicle. The bones most subject to this disease are the metacarpal bones and the fingers: in fact, five-sixths of the cases of enchondroma in the bones have occurred in those parts. Three cases were observed in the leg, one only in the thigh, one in the os ilium, one in the basis of the skull, and one in the ribs.

### 2. *Different Forms of Enchondroma in the Bones.*

Enchondroma affects two forms—one, the more common, a central one, developed in the interior of the bone, and accompanied with expansion of the osseous shell—the other, on the surface of the bone, unattended by expansion of the bony shell.

A. *Enchondroma of the Bones with Osseous Shell.*—This is by far the more frequent form in the smaller cylindrical bones, in the metacarpal and metatarsal bones, and in the phalanges of the hand and foot. The first changes are softening of the spongy substance in the interior of the bone, the place of which is occupied by the soft mass of enchondroma. While this process is going on, the shell of the bone becomes dilated, as though from the action of some internal force. This distention, however, can be the result only of some change in the nutritive process, for the attenuated shell does not break, but long maintains its perfect continuity. In proportion as the old bone is destroyed, new bone is deposited on the surface of the tumor, and thus the shell of the bone is the subject of a constant alteration. The progressive growth of the new substance in the interior of the bone dissolves here and there the continuity of the bony shell; and in course of time this shell is reduced to a few thin isolated laminæ on the surface of the tumor, which still retains its smoothness, and spheroidal shape.

It seems dubious whether Sir A. Cooper has described this form of enchondroma under the head of cartilaginous exostosis of the medullary membrane. M. Müller, however, expressly cautions us against confounding fibrous tumor from the interior of the bone with enchondroma.

B. *Enchondroma of the Bones, without Osseous Shell.*—In some bones in which the spongy tissue predominates, enchondroma occasionally becomes developed from their exterior without being invested with a bony shell—as from the pelvis, cranial bones, and ribs. The tumor is, in these instances, less regularly spheroidal, and its surface less smooth, displaying an agglomeration of roundish bodies, some larger than a pea, others smaller, which are cells containing a soft, grey, cartilaginous mass. The whole tumor is made up of a conglomeration of such cells. The preparation in the museum of the College of Surgeons, in London, labelled, "cartilaginous tumor formed on a man's ribs," is of this nature. The museum of Saint Bartholomew's Hospital contains (Morbid Preparations, eighteenth series, No. 14) a specimen of enchondroma of the basis of the skull. In the museum of the Middlesex Hospital is an immense mass growing from the inner surface of the ilium, which presented the structure of enchondroma.

The larger cylindrical bones are also sometimes liable to the exogenous form of enchondroma, especially if that growth is developed in parts where the spongy structure abounds, as in the upper end of the tibia. In such a case, the tumor may be destitute of osseous shell. A preparation of this sort (Morbid Preparations, first series, No. 41) is contained in the museum of Saint Bartholomew's Hospital.

The museum of Guy's Hospital contains a preparation, numbered in the catalogue 666*, and called an osteo-sarcoma. It is an enchondromatous growth, without a bony shell. Sometimes this growth appears on the leg, encased within a bony shell, at least the examination of two dried preparations in the museum at Berlin seems to warrant this conclusion. In some rare cases of enchondroma of the phalanges of the finger, the tumor is destitute of bony shell, as may be seen in two preparations of enchondroma, in the museum of Guy's Hospital. M. Müller thinks it evident that what Sir A. Cooper describes as cartilaginous exostosis between the periosteum and bone is only ordinary external osseous exostosis with cartilaginous animal basis.

### 3. *Microscopic Examination of Enchondroma.*

The minute structure of enchondroma corresponds exactly with that of cartilage, and all that the employment of high magnifying powers has brought to light with regard to the latter substance, has been confirmed by examination of the former. But it resembles more the cartilage in the embryo than in the adult. In most cases cells with nuclei only are seen ; the presence of secondary cells is more rare. Usually the cells are in close contact with each other, and there is no sign of development of intercellular substance, though, in a few cases, a clear substance may be distinguished between the cells. Here and there bundles of fibres are visible.

"The size of the cells," continues M. Müller, "exceeds several times that of the red particles of human blood. The nuclei, which have a diameter of 0.00032 to 0.00042 of an English inch, are sometimes round, at other times oval or irregularly elongated. The nucleus appears somewhat flattened, but its form is often very irregular. In addition to the nuclei, corpuscules are seen here and there, of an irregular form, furnished with spiculated appendages, often of considerable length, and similar to the spiculated osseous corpuscules described by the author. Occasionally these spiculæ extend over a whole cell, or even further.

In most instances, enchondroma remains stationary at that stage of development which cartilage attains in the embryo, and presents an almost entirely cellular structure. A very firm and hard cartilaginous tumor of the testicle, however, which once came under the author's notice, showed a very high degree of development in the intracellular cartilaginous mass, and closely resembled the natural appearance of those cartilages in which the cellular structure is not permanent." 109.

### 4. *Chemical Analysis of Enchondroma.*

A. *Enchondroma of the Bones.*—If, says M. Müller, portions of enchondroma of an osseous structure are boiled for ten or eighteen hours, they yield a considerable quantity of gelatine, which forms a jelly immediately on cooling, but differs completely in its chemical characters from ordinary gelatine, or *colla* ; while it coincides exactly with the peculiar gelatine which the author has discovered in cartilage, and to which he has applied the name of *chondrine.* The author detected it first in enchondroma, afterwards in the permanent cartilages. M. Müller proceeds to offer a description of these two sorts of gelatine.

1. *Colla,* common glue, the gelatine obtained from tendons, membranes, bones. The characters of this kind of gelatine are well known, and it is also a fact familiar to all, that isinglass differs from ordinary gelatine, or glue, only in being more soluble in alcohol. Its behaviour to the common chemical reagents we need not stop to consider.

This kind of gelatine is extracted, by boiling, from the skin of man or of beasts, from tendon, from fibro-cartilage, from the inter-articular cartilages, from cellular tissue, serous membranes, and from the cartilages of bones after they are ossified ; but is not obtained from the permanent cartilages, nor from those of the bones previously to their ossification. The same kind of gelatine was likewise obtained by boiling from enchondroma of the parotid gland : enchondroma of the bones, however, and of the testicle, became resolved by long boiling into cartilage gelatine, or chondrine.

2. *Cartilage gelatine, chondrine.*—This matter exists in the permanent cartilages, with the exception of the tendino-fibrous cartilages; in the cartilages of the larynx, in those of the ribs, and of the joints, and in the cornea ; all of which yield it if boiled for ten, fifteen, or eighteen hours, and become wholly converted into it if boiled for a sufficiently long time. A concentrated solution of chondrine is less coloured than a solution of common glue. A solution of it, like a solution of common glue, solidifies in cooling, and forms a clear jelly ; if evaporated to dryness, the matter is less brown than ordinary glue. Though chondrine is capable of forming a jelly, though it swells up when moistened with cold water, and is dissolved by hot water, just like ordinary gelatine, and though infusion of gall nuts, chlorine, alcohol, and corrosive sublimate produce the same results with both, yet the former is differently affected by alum, by sulphate of alumina, by acetic acid, acetate of lead, and sulphate of iron. All these matters precipitate chondrine, while they do not produce the slightest effect on ordinary gelatine. These precipitates are not soluble either in hot or in cold water, though they may be dissolved by an excess of the solutions of alum or of sulphate of alumina, which ought therefore to be added only drop by drop to any fluid from which it is wished entirely to separate the chondrine. Evaporation of the filtered fluid will shew when the chondrine has been entirely separated; for the fluid then ceases to gelatinize, and, indeed, contains only a minimum of animal matter. Hence it is evident that the presence of chondrine is the cause of the gelatinization of a solution of permanent cartilage, and that chondrine is not contained in it in addition to gelatine, as a sort of subsidiary constituent. We have not space for the elaborate account of the chemical properties of chondrine furnished by M. Müller, nor for the distinctions between it and caseine. Nor can we pursue our author in his hypotheses on the exact relation between colla and chondrine, and on the causes of their differences. It is certainly true, as he remarks, that among the most remarkable chemical changes which any tissue undergoes, are those which ossification produces in the cartilages of bones. According to the author's observations, cartilage becomes changed during this process from chondrine into gelatine, and this metamorphosis takes place alike in healthy and in morbid ossification. The cartilages of the ribs, larynx, and trachea, and the cartilaginous investments of the articular surfaces, become resolved into chondrine by boiling during fifteen or eighteen hours. The cartilages of unossified bone yield the same substance; but bones, when ossified, do not contain chondrine, but only gelatine. In performing these experiments, it is a matter of indifference whether or no the cartilage is freed from the salts of lime, by means of hydrochloric acid, before boiling; for in either case the gelatine obtained is the same, and resembles common glue.

It is evident, he goes on to say, that a great change takes place in cartilage gelatine at the time of the ossification of bones; whether owing to a change of some of its constituents, or to its combination with new ones, as salts; phosphate of lime for instance. This change seems to be essentially necessary to ossification, for, as far as is yet known, no bone, when ossified, contains an appeciable quantity of chondrine. Even the permanent cartilages lose it during accidental or morbid ossification.

The morbidly ossified cricoid and thyroid cartilages of the human larynx were examined, such parts as preserved their cartilaginous structure being carefully separated before boiling. The gelatine obtained by pounding and boiling the ossified parts was not chondrine, but colla; it was not precipitated by acetic acid, alum, sulphate of alumina, or acetate of lead. The two former reagents produced a few scarcely perceptible isolated flocculi, which were seen only on looking at the fluid with close attention. Since, however, they immediately precipitate chondrine, these flocculi were doubtless traces of it arising from some imperfectly ossified portions of cartilage. A permanent cartilage, which, as such, contains chondrine, changes that substance (either before morbid ossification begins, or while that process is going on) into bone, gelatine, or colla.

It was, reasoning on these facts, not unnatural to suppose that diseased bones which have lost, more or less completely, their salts of lime, would give out chondrine instead of colla. Such, however, is not the case.

"The alteration which the animal matter undergoes in osteomalacia is of a peculiar kind. The author has examined softened bones in the human subject and in other animals. In both cases, long-continued boiling produced neither colla nor chondrine. The extract continued thin and fluid, did not gelatinize when evaporated, passed turbid through the filter, and, when a finer filter was employed, was seen to be transparent, and of a yellowish-brown colour; it was precipitated by tincture of galls, and by alcohol, but neither by acetic acid, acetate of lead, or sulphate of iron. Sulphate of alumina did not cause any marked precipitate, but threw down only a few flocculi, which were not seen except by looking attentively, and which were dissolved by adding sulphate of alumina in excess. Liquor potassæ caused a precipitate. It should be observed that the author speaks here only of the highest degree of osteomalacia, for the bones he examined were perfectly soft and pliable. The spiculated osseous corpuscules are still visible in such bones, although the matter of which they are composed has evidently undergone a peculiar change. In the case of a goat, the pliable pieces of bone became brittle when boiled for a long time, and the water was rendered turbid, and was mixed with much fat. Portions of the os calcis of a man affected with osteomalacia were still softer, and contained a large quantity of fat in their spongy substance: they were boiled in alcohol to extract the fat, and the structure which remained was membranous, pliable, rendered softer by long boiling, but did not swell. It appears that the cartilage in osteomalacia becomes softened by changes in its constituents, or by combinations with salts, and that a substance remains which may be partially extracted by boiling, but which does not gelatinize."

"This examination shews how great is the difference between osteomalacia and those changes in the bones which enchondroma produces. In true softening of the bones, their gelatine completely loses its nature ; in enchondroma, on the contrary, there is a new primitive formation of cartilage, exactly similar to its first formation in the embryo; its substance, therefore bears no chemical resemblance to such as have become ossified, but is real chondrine. On boiling the contents of a very remarkable variety of enchondroma of the bones, the author extracted a quantity of matter which gelatinized completely when cold; but this jelly was chondrine, for its solution was precipitated by alum, acetic acid, acetate of lead, and sulphate of iron ; and a few drops of a solution of alum precipitated all the gelatine from a considerable quantity of fluid in large masses, which were not redissolved in hot water. In this disease permanent cartilage had become developed, with morbid growth in the interior of the bone." 124.

B. *Enchondroma of the Soft Parts.*—Different specimens furnished different results. A very firm cartilaginous tumor of the testicle, which had developed itself in an elderly man, close to a carcinoma reticulare of the same organ, but perfectly unconnected with it, and in which the cartilage cells were separated by the intervention of a firm substance, yielded chondrine when boiled. On the other hand, not only could no chondrine be obtained from the enchondromatous growth of the parotid gland, but the tumor gave out readily a large quantity of gelatinizing colla.

### 5. *History of the Development of Enchondroma.*

Our author first notices the development of Enchondroma, as observed under the microscope.

Schwann has proved that the cartilages of all animals are originally cellular in the embryo, and he established the principle of their formation from the nuclei of cells, and discovered how young cells are developed within the cavity of older ones.

Now, without going into the details of the microscopic development of enchondroma, we may cite the general conclusions with respect to it.

" The chief difference between the morbid and the natural formation of cartilage consists in the persistence, in the former, of that cellular structure which exists in the embryo. Many other tumors afford illustration of this remark. It is not any peculiar form of their elements which stamps upon morbid growths their distinctive character, but it consists partly in the formation of the ordinary primitive structures, where they are not necessary; partly in the imperfect development of these tissues, and its arrest at a stage which, in health, is but transitory. In the healthy primitive formation of cartilage, the vital principle of the whole organism controls the monad state of existence of the cells, and sets to it bounds which it does not pass. In process of time the walls of the cells thicken, and an interstitial indistinctly fibrous mass of cartilage is formed betwixt the cavities of the germinal cells. In enchondroma, on the contrary, the sunken vitality of the part in which the diseased growth is developed seems to set to it no such limits, but the growth proceeds, slowly increasing to a larger and larger size. Usually the walls of the cells do not thicken; the formative process cannot raise itself above that form of cartilage which first exists in the embryo, but continues without ceasing to reproduce this embryonic structure." 127.

From the microscopic development of enchondroma, M. Müller passes to the duration and termination of enchondroma.

The great length of time that these tumors may exist, is shewn by the history of many cases of this disease which have been erroneously regarded as cancerous. M. Müller refers to several recorded cases in proof of this. In all these cases, the long time, often fifteen or eighteen years, occupied by the disease in its development, and the absence of dangerous symptoms during that period, are very striking. All writers too, concur in representing the development of these tumors as being unattended by much pain. So that enchondroma may continue long, occasion little suffering, and give rise to no degeneration of the superincumbent parts.

But, when inflammation is set up in the tumor, it becomes painful, and bursts. The distention of neighbouring parts, and accidental injury to the enormous tumor bring on, in the course of time, inflammation of it and of the neighbouring textures; inflammation is followed by suppuration, the tumor itself discharges sanies, and the bones, the proper structure of which is already destroyed, become necrosed. Such was the state of the disease in the cases described by Mery and Scarpa.

If the part affected by the tumor is amputated, the disease does not return; but if, after the tumor has burst it still remains in connexion with the body, it may, like every extensive local disease, bring with it the ruin of the whole constitution.

### 6. *Nature of Enchondroma.*

It has been already stated that enchondroma consists mainly in the formation of cartilage resembling the primitive form of that substance in the embryo. Its causes are partly local, partly general.

*Local Causes.*—These may be stated, with great certainty, to be serious injuries to the vitality of the bones, and, in many instances, mechanical violence. This is confirmed by the cases already related, and will be found to be no less substantiated by the histories of other tumors which older surgeons have written, and in which, though the names applied to them are often very various, yet the characteristic features of enchondroma may be easily distinguished.

Severinus quotes a case from Nicolaus Larcho, in which the tumors had appeared in consequence of the bite of a pig, while the patient was young; and in the cases of Schaper, Otto, Klein, and Ph. v. Walther, the disease was referred to a bruise.

*General Causes.*—In some instances no local causes can be made out, and there is strong evidence of the existence of some powerful general one. A case of Professor Pockels is in point. The tumors of the metacarpal bones and phalanges of the fingers had formed not on one hand only, but there was a commencement of the same disease in the other hand; and the most singular fact of all was, that the feet shewed a disposition to become the seat of the same morbid procees. The pathological changes in the feet and in one hand were but slight, and did not occasion inconvenience, but the other hand was amputated. The disease did not return; the patient is still alive; his hand and feet are in the same state as before, and probably there is nor much reason to dread the further development of the disease, since it had begun in the earliest infancy of the patient, and had progressed very slowly. Ruysch, likewise, has described some cases in which tumors grew from the fingers and metacarpal bones of both hands, and from the toes of both feet.

M. Müller thinks this general cause akin to scrofula. Both diseases are constitutional, but in no way related to carcinoma, and both are most active during childhood. The observations which have already been adduced suffice to prove that enchondroma occurs most frequently during childhood. Most of the persons in whom it was seen were young men and boys, in whom it had arisen at a very early age. In after-life the general predisposition to enchondroma seems to be extinguished, just as is the case with scrofula. The tumors, which are the effects produced by the previous disease, continue to exist and cannot be removed; but in the adult, after the diathesis has ceased, they continue as merely local affections, which, therefore, do not return after amputation. Yet there are distinctive differences between enchondroma and scrofula, for the affections of the bones in the latter are well known and peculiar, nor do scrofulous growths and tubercles occur in enchondroma. The cause of enchondroma, says our author, seems to consist rather in a peculiar formative process in the osseous system, in consequence of which, especially when local injuries have been inflicted on the bones, cartilage, in its primitive embryonic state, is developed, and continues to be formed without ever attaining to consolidation or perfect organization. The natural tendency of the part to ossification, and the influence of the whole organism, are unable to control the growth of enchondroma, which is produced by the innate vitality of the cartilage cells, and their unceasing multiplication. Other alterations of the bones are very rarely combined with enchondroma; indeed, the only instance of it with which the author is acquainted, is that of a hump-backed man, whose case Severinus has related.

Enchondroma has figured under various names. It has been successively "Spina Ventosa," (one of many affections lumped together under that ridiculous denomination,)—"Atheroma Nodosum,"—"Osteo-Steatoma,"—"Osteo-Sarcoma." Otto has confounded it with cancer of the bones, how erroneously the following cheering statements will shew;—

"The amputation of enchondroma is almost invariably successful, as in the cases of Severinus, Mery, and Scarpa, in the two cases related by Ph. v. Walther, in the two which occurred to Klein; in the case of enchondroma of the tibia, the preparation of which is now in the Museum of St. Bartholomew's Hospital; in the case observed by Professor Pockels; and in those which came under the notice of Professor v. Graefe, the preparations illustrating which are now in the museum at Berlin. Of thirty-six cases, the histories of which have been ascertained by the author, two only had a fatal termination: in one of these cases, the tumor, which was situated on the base of the skull, encroached upon the cavity of the cranium, as well as on that of the nose, and proved fatal, owing to its situation. The second case has reference to an enchondromatous tumor of the thigh bone, which is now preserved in the museum of Guy's Hospital, and is numbered 666* in the catalogue. In this instance, the tumor, which probably brought on death by the loss of the fluids which it occasioned, was removed from the body of the patient after he was dead."

### 7. *Differences between Enchondroma and other Tumors of the Bones.*

Enchondroma has no resemblance in structure to schirrhous and medullary tumors of bones, *their* basis being albumen, and that cartilaginous mass, which, when boiled, yields chondrine, being absent.

If fungus medullaris is developed in the interior of a bone, it neither perforates it nor expands it in a spherical manner. It is only in very rare cases of medullary sarcoma that real expansion of the bones takes place. If fungus medullaris is developed on the surface of a bone, it often contains in its interior delicate acicular, or lamellar spiculæ, which shoot from the surface of the bone. In cancer alveolaris, cavities may be observed filled with a transparent jelly; but this structure differs both microscopically and chemically, and, when boiled, yields no gelatine.

"The bones," continues M. Müller, "are subject to another fungous growth, which differs not less widely from enchondroma, and resembles it only in its curability by amputation. The author had an opportunity of studying the characters of this growth, the tumor fibrosus s. desmoides, on a hand which was extirpated with perfect success, by M. v. Graefe. The tumor projected from several of the metacarpal bones, both towards the palm and towards the back of the hand, and presented a lobulated surface and a firm tendinous structure in its interior. When divided, it shewed that white and perfectly fibrous structure whence the tumor derives its name, and which similarity to the glistening satinny tissue of the aponeurosis is characteristic of the desmoid growths. Under the microscope it displayed layers of fibres intertwined, without any trace of cavities or corpuscules. Its base was seated on the surface of the metacarpal bones, from the periosteum of which it was developed; while the bone beneath remained undestroyed, and only slightly rough, as it always is in the neighbourhood of tumors. The arteries of the palm, the muscles, and tendons, ran in the form of an arch over the tumor. The museum of St. Bartholomew's Hospital contains a specimen (Morbid Preparations, first series, No. 148, 149,) of this disease in the substance of the lower jaw. The tumor had developed itself in the interior of the bone, and likewise on its surface."

Enchondroma cannot be confounded with the osteöid, or purely osseous tumor of bone.

But there are other growths, curable by amputation, though often confounded, under the names of osteo-sarcoma and osteo-steatoma with cancer of bone. That form of morbid growth called osteosarcoma, which is not rare in the bones of the face, and particularly in the lower jaw-bone, is a fungus of a peculiar nature, curable by amputation. Its substance never resembles cartilage, is of a greyish white colour, of an albuminous nature, and cannot be resolved into gelatine by boiling. Examined under the microscope, it presents a structure composed of minute cells furnished with nuclei, or else a soft tissue made up entirely of caudate corpuscules, whose linear arrangement gives it a fibrous appearance. These osteosarcomatous growths are sometimes composed entirely of an albuminous substance, but in other cases they yield some gelatine after long boiling. It is moreover, far from an easy matter to distinguish between these growths and those tumors of the bones which are really of a cancerous nature. This latter is an important observation, and should not be forgotten by those who affect the possession of certain means of diagnosis between the malignant and the non-malignant tumors of the facial bones.

Expansion of the bones is sometimes occasioned by the development within their substance of compound cysts, or of hydatids. These constitute a destructive disease, which sometimes implicates a great part of the osseous system. Hydatids are formed in the medullary tissue of the bones, and in other cases they do not produce this effect. The spongy bones, as those of the pelvis, or the ribs, are most subject to expansion; and when it does take place, the hydatids are usually found lying in a soft bed abounding in fat, which is a growth from the medullary texture; while the osseous tissue is absorbed, and forms only fragments here and there in the interior of the tumor. The shell of the bone becomes distended in a spherical manner, as in enchondroma. The disease, as might be expected, renders the bones very liable to fracture, and it often leads to a fatal result.

M. Müller devotes a section to the cases of enchondroma on record, and to the specimens contained in anatomical museums—a very useful section, and we recommend it to the reader. We have not, however, space, nor is it necessary for our purpose to notice it. We therefore pass to:—

### II. ADIPOSE TUMORS.

The cells of adipose tissue are shut sacs, within which the fat is contained. In most animals these sacs are round, but in some, as the sheep and ox, they have a polyedrous form. It is more difficult to detect the nucleus in the wall of adipose cells, than in cells of other tissues; but, even in them, Schwann has demonstrated the existence of nuclei.

Morbid adipose tumors are repetitions, more or less modified, of normal adipose tissue. Some are made up of ordinary fat, such as is found in the healthy adipose tissue of the human subject, while others contain likewise cholesteatoma, and may be distinguished by their laminated structure.

M. Müller divides adipose tumors into three classes:—The varieties of lipoma form the first; adipose cysts the second; and the laminated fatty tumor the third. In lipoma the fat is contained in ordinary adipose tissue, and is consequently separated into innumerable isolated compartments by the walls of the contiguous cells. In adipose cysts, the fat is not distributed through small cells, but is contained, partly in a fluid state, partly in the form of fat globules, in the interior of a larger sac, which is generally furnished with thick membranous parietes. In the former, the production of the new fat takes place just as in the healthy body; while in the latter a single fat cell appears to become predominant, and, its walls being thickened, it constitutes an independent cyst.

1. *Lipoma.*—Most specimens of lobulated lipoma resemble in structure ordinary adipose tissue; their cells are of a round or oval shape, and the only distinction between the two consists in the former being made up of a mass of these adipose cells, enclosed by an investment of thickened cellular tissue, while the different lobuli are separated from each other by thinner membranous septa.

M. Müller arranges lipoma thus:—

A. *Lipoma Simplex,* to which the name lipoma is usually applied. These growths seem capable of being formed in any part where there is cellular tissue. In Meckel's museum at Halle, the author saw a small adipose tumor which was situated between the optic nerve and eminentiæ candicantes.

B. *Lipoma Mixtum.*—In this case, the interstitial cellular tissue of the adipose tumor is greatly developed, and forms strong membranous septa, which intersect its substance, and thus give it a much greater degree of firmness than is possessed by simple lipoma. Hitherto the author has met with but two specimens of this form of tumor; once in the spermatic cord of a man, and once between the muscles of the thigh; and in this latter case the tumor had acquired an extraordinary size.

C. *Lipoma Arborescens.*—This structure consists of arborescent productions, entirely composed of adipose tissue. Growths of this sort occur in the joints, especially in the knee joint, where they spring from the free portion of the synovial membrane. In this situation they are covered by a prolongation of the synovial membrane, and hang loosely into the cavity of the joint, forming arborescent tufts, somewhat swollen at their extremities.

### 2. *Adipose Cysts.*

The ovaries are the most frequent seat of this disease, in which fat, partly in a fluid state, partly in the form of globules and free from adipose cellular tissue, is contained in a large cyst, with dense parietes. In addition to fat, these cysts usually contain hair, either loose within their cavity, or proceeding from their walls; each hair springing from a distinct follicle. In birds, feathers are found in these situations. Gurlt regards the fat in these hair-containing cysts as analogous to that which is formed by the sebaceous follicles of the cutis, and poured by them into the hair follicles. The sebaceous follicles of the skin may themselves become converted into cystoid tumors, and to the accidental occlusion of their orifices many encysted tumors owe their origin. This fact had been rendered probable by M. v. Walther and Sir A. Cooper; and in one instance the author met with a decisive proof of its truth. In an individual, all the sebaceous follicles of the skin of the nose had become extremely enlarged, and one was converted into an encysted tumor eight lines in diameter. Microscopic corpuscules of irregular polyedrous shape, and resembling epidermoid or epithelium cells, except in being destitute of nuclei, formed the contents of these cysts. These corpuscules are probably produced in a manner similar to epithelium cells.

### 3. *Laminated Adipose Tumor—Cholesteatoma.*

This is a non-lobulated tumor, composed of concentric layers of polyedrous cells, which have a lustre like mother of pearl. It is of the consistence of tallow, and is usually invested with a thin membrane which forms a complete cyst. The fat which it contains is not found exclusively within the cells, but is likewise deposited in their interstices. This form of adipose tumor, was formerly but little, if at all known; and M. Cruveilhier was the first who examined its nature with the attention which it deserves. M. Müller refers to growths described by Dr. Merriman, M. Lepestre, M. Cruveilhier, and M. Dupuytren, which he regards apparently with justice as instances of cholesteatoma. In Dr. Merriman's case, the tumor was situated between the rectum and cervix uteri—in M. Lepestre's, it had developed itself in the brain—in M. Cruveilhier's (two) the brain was also the seat of the disease—and M. Dupuytren saw fatty matter surrounding some carious vertebræ in the cancellous structure of the lower jaw, and lining some old urinary fistulæ. M. Müller describes it methodically.

A. *General Structure of Cholesteatoma.*—The mass is soft semi-transparent, of the colour of white wax, but glistening like mother-of-pearl. It shrinks much when dried, and at the same time loses its whiteness, and becomes of a yellowish-brown colour, though it still retains somewhat of its glistening appearance. It is usually made up of laminæ no thicker than the finest paper, which are in most instances arranged in a concentric manner, as the author has had an opportunity of observing in tumors of the cerebrum. The tumors are generally round, or oval, or of an uneven though rounded form, and on their surface are seen those small nodules represented by Cruveilhier, the layers of which have also a concentric arrangement. Sometimes fragments of a regularly laminated structure are irregularly intermixed, like broken masses of some stratified rock. Such was the nature of a cholesteatoma in the interior of the cranial bones, the external table of which it had caused to bulge outwards, while it had destroyed the internal table. A distinct membrane enveloped this mass, and, indeed, all the tumors of the brain and the cranial bones were covered by a very delicate membrane. There are cases, however, as will hereafter be shewn, in which cholesteatomatous matter is deposited in the interior of cysts with thick parietes, or upon the surface of ulcers,

The laminæ are easily separable with the point of the scalpel, The surface of sections made perpendicular to the laminæ does not present the mother of pearl lustre.

B. *Microscopic Structure.*—The finer elements of cholesteatoma are a cellular tissue, composed of very minute polyedrous cells, which constitute the laminæ, and a crystalline fat deposited in the interspaces between those laminæ.

"This cellular tissue bears no resemblance to the adipose celular tissue which exists naturally in the human subject. Its cells are completely polyedrical, like many pigment cells, and it is exactly analogous to the cellular tissue of plants. It is somewhat similar to the polyedrous cellular tissue of the fat of the sheep, but the cells of cholesteatoma are more than twice as small. The average diameter of the cells of cholesteatoma is 0.00081 of an English inch. The cells are as irregular in form as those of the fat of the sheep, some of them being pentagonal others hexagonal, and almost all of them having unequal sides, which irregularity in form is produced by the cells being in contact with each other. A few cells of a more regular, almost dodecahedral form, are occasionally seen. The laminæ, which may be detached from each other with the point of the knife, far exceed in thickness the diameter of a single cell; consequently, by changing the focus of the microscope, other cells are brought into view. The separation of these tumors into laminæ is probably the result of the successive formation of cells and of the deposit of crystalline fat in their intervals.

The individual cells are easily separated, and, when isolalted, they are seen to be very transparent and pale, not containing a nucleus in their wall or cavity, nor a finely granular substance in their interior. It is indeed very difficult to detect any wall to these cells, and consequently there is some doubt whether these bodies are solid or hollow. But the fat cells of the sheep present, when isolated, exactly the same difficulty, although there is no doubt as to their being furnished with walls.

With regard to the substance of the cells, their basis is formed by an animal matter quite distinct from fat. If portions of a cholesteatomatous growth are treated with boiling alcohol or æther, the greater part will remain undissolved, will still retain its laminated arrangement, and will present, although less distinctly, its cellular structure. The same may likewise be observed on heating the substance upon a plate of glass; from which too it acquires, like all animal matter, a brownish tinge. It is probable that a part only of the fat which may be extracted from these growths is contained within the cells.

The crystals situated between the layers of cells are of two kinds, tabular and lamellar; and both forms are easily distinguishable by means of a microscope. The tabular are the most numerous; they are scattered in all directions, and here and there some of them are broken. Their length differs much, in proportion to their breadth; they are often short, broad, and rectagular; but frequently ribbon-like, long and narrow, far exceeding in length the diameter of the cells, and being then very easily broken. The shape of some of the tables seems to be rhombic; but it is not easy to say whether this is really the case, since rectangular tables, if lying obliquely, would resemble rhombs. These tables are not acted on either by acids or alkalies, and probably consist of pure cholesterine. Pure cholesterine, which the author examined under the microscope, was composed almost entirely of rhombic tables.

The other crystalline bodies which the author saw are less abundant, and are collected here and there into little bundles of lamellæ, which, when seen edgeways, might be taken for aciculæ of stearine. If treated, however, with boiling alcohol or æther, it will be seen, as they are deposited on cooling, that their real form is that of lamellæ, pointed at both ends." 160.

C. *Chemical Relations.*—From experiments by M. Barruel it appears, that the portions of cholesteatoma not dissolved by alcohol, possess all the characters of albumen, while the alcoholic solution is made up of cholesterine and stearine.

D. Of the *Seats and Forms of Cholesteatoma.*—All parts of the body appear to be liable to cholesteatoma. It has been met with twice in the substance of the bones; the author saw it once in the bones of the skull, and M. Dupuytren met with a growth probably of this nature in the lower jaw: he also observed it around some carious vertebræ. Leprestre saw it once in the brain, Cruveilhier twice, and the author three times in that situation. When in the brain, it is situated either on the surface of that organ, as in the cases which occurred to M. Cruveilhier and the author; or in its substance, as in the case seen by Leprestre. Dr. Merriman met with it between the uterus and rectum. The author saw it once in a cysto-sarcomatous growth of the mammary gland, and thrice in a subcutaneous cyst. This tumor is most frequently observed in the brain, for, of sixteen cases, six occurred in the brain, three in the bones, and two on the surface of ulcers.

When this morbid growth forms in the bones, it distends and sometimes bursts their shell, a fact which Dupuytren was witness to in the lower jaw, and the author in the cranium.

The forms are either cysts, or on the surface of ulcers.

*a, Cholesteatoma in Cysts.*—This seems the prevailing form. In internal parts, as in the brain, the cyst is delicate—beneath the skin, as might be expected, it is thicker.

*b. Cholesteatoma on the Surface of Ulcers.*—Of this there are two instances, one of which came under the observation of M. Dupuytren, who found cholesteatoma lining urinary fistulæ, and the other case was seen by the author. The author found, on examining with the microscope, a cancerous ulcer of the female breast, that its surface was covered with a layer of a peculiar matter, such as he has never seen, either before or since, in that situation. This substance was composed entirely of the fatty matter of cholesteatoma, and presented the ordinary structure of nucleated polyedrous cells.

E. *History of the Development of Cholesteatoma.*—"It may be assumed," says our author, "with tolerable certainty, that cholesteatoma is destitute of bloodvessels; for not only have they never been seen, either by the author or by any others who have directed their attention to the subject; but, likewise, the growth of a cholesteatomatous mass by aggregation of successive laminæ within cysts, as in cholesteatoma cysticum, suffices to prove that the possession of vessels is not essential to its formation. It must, then, be formed in a manner similar to the yoke cells within the cavity of the yoke bag, and, like the epithelium cells, its increase must take place by the deposit of successive lamellæ. So close, indeed, is the resemblance between the epithelium cells and those of cholesteatoma, that they coincide in all points except in the circumstance that the latter are destitute of a nucleus. The cells of ordinary adipose tissue do, therefore, present less resemblance in the manner of their formation to cholesteatoma than is shewn by the horny structures on the surface of the skin; for the walls of adipose cells are furnished with bloodvessels, which may be demonstrated by minute injections. This apparent difference, however, diminishes on a close examination of the subject; for the original genesis of all cellular structures takes place independently of bloodvessels, in a manner like to the formation of vegetable tissues. This is illustrated in the structure of epidermis and epithelium; the newly-formed cells removing further and further from the seat of their formation, and losing their vitality, in proportion as new cells are produced in the more deeply-seated layers of the epidermis. In the present state of our knowledge the formation of epithelium and of the yolk cells best illustrates the origin and growth of cholesteatoma. It is not probable that the cells of cholesteatoma continue, when once formed, to vegetate like those of other structures, and to produce new cells; for the author never observed in this form of morbid growth young cells encased within older ones, nor any thing approaching to the relation between parent cell and germinal cellule. The cells, when once formed, are moved by the generation of new ones from the place of their original formation, and thus successive laminæ are formed, as in all epithelium structures." 164.

F. *Nature of Cholesteatoma.*—It is not malignant. When occurring on the surface, or in the substance of an ulcerated carcinoma, it is an accidental complication of that disease. Many cases can be adduced in proof of its not returning after extirpation.

M. Müller refers to seven preparations of cholesteatoma in the museums of Berlin and Halle.

### III. Of Compound Cystoids and Cysts. Sarcomatous Growths.

### 1. *Of Compound Cystoid Growths.*

M. Müller refers in terms of commendation to the well-known observations of Dr. Hodgkin on compound serous cysts. Our readers are aware that, according to the doctor, the compound cysts are of two kinds. In those of the first, new cysts develop themselves in the walls of the old one, without growing especially in the direction of its cavity, and without being attached to it by peduncles. A repetition of this process forms a tumor composed entirely of cysts of different sizes.

In the second kind, the new cysts are attached by pedicles to the walls of the parent cyst, into the cavity of which they project. These pyriform growths may proceed from one or from several parts of the parent cyst, and may themselves give rise to tertiary growths. Dr. Hodgkin has detailed the history of this structure with great minuteness. The inner membrane of the parent cyst is reflected upon those of secondary formation. These secondary pyriform pedunculated cysts do not contain merely a serous or mucous fluid like that of the parent cyst, but within them are clusters of new growths, which proceed from one or more points of their internal surface, and occupy their cavities. Sometimes the young cysts are attached to the older ones by a thin stalk; at other times they have a broader basis. Sometimes the quantity of fluid contained in the parent cyst is great in proportion to the size of the secondary cysts which sprout from its walls; in other cases the opposite occurs, and the dilatation of the secondary cysts may go so far as not merely to distend the parent cyst, but even to occasion its rupture. The secondary cysts, too, may be ruptured, and then they appear like follicles pouring their contents into the parent cyst. The membranes of these cysts are susceptible of inflammation, which may proceed so far as to cause the adhesion of the secondary cysts to the parent sac. Suppuration, likewise, may take place in these cysts, and, consequently, pus may be found in their cavities.

M. Müller speaks to the correctness of these observations of Dr. Hodgkin's. But he cannot coincide with him " in his attempt to extend the principle of the formation of compound serous cysts to explain the structure of sarcomatous and carcinomatous tumors. The principle of the development of these latter tumors is, as observations with the compound microscope shew, perfectly different in its character. Their ultimate elements, indeed, are cells, often permanent, so small as to be distinguishable only by employing a high magnifying power; but these cells were never observed by Dr. Hodgkin. These cells, moreover, do not sprout from the parietes of a parent cell, but are developed around nuclei, which are formed either free in the interior of the parent cells, or external to their cavity. Their development does not differ from the development of the tissues in the embryo.

The substance of the walls of a compound cyst is firm, white, often possessed of a satinny lustre, and is composed of regular layers of fibres. The contents of the sac are sometimes a clear fluid, like mucus, at other times they are formed by a pulpy and turbid matter. If tumors of this kind are preserved in spirits of wine, tabular and rod-shaped crystals may be found in the matter which they contain, as well as granules resembling small vesicles filled with a minutely granular substance.

It is worthy of notice, that the clustered growths which proceed from the interior of compound cysts with endogenous development are, often, almost or altogether solid. Instances of this have frequently come under the author's notice; for instance, in ovarian cysts which were beset internally with clusters of sprouting growths. The interior of these growths was, indeed, soft, but presented no trace of cysts; instead of which it was partly fibrous, and, on a high magnifying power being employed, cells were discovered, such as are found in firm sarcomatous growths; the author, therefore, does not think that the process of development described by Dr. Hodgkin invariably occurs. The same observation has likewise been made by Sir A. Cooper,* with regard to the pedunculated growths sometimes contained in the interior of hydatids of the female breast; for he says that some of them were composed of a cellular tissue, infiltrated with fluid. The converse of the ordinary process may, therefore, sometimes take place, and the pyriform bodies, being first produced, may either remain solid, or become the seat of the development of cysts."

Cysts sometimes contain fungous growths, which proceed from the interior of their parietes.

### 2. *Of Cysto-sarcomatous Growths.*

The author applies the name of cystosarcoma to such tumors as, while they are principally composed of a more or less firm, fibrous, and vascular mass, are yet invariably found to contain solitary cysts in their substance. The fibrous masses are composed of an albuminous substance, and sometimes contain granules scattered between their fibrils. A person regarding merely the nature of the mass which forms the bulk of the growth, would unhesitatingly refer it to the class of sarcomata; but the presence of cysts is so invariable, and these tumors so often exhibit peculiar forms, as to afford just grounds for forming them into a new species, which, indeed, is rendered more desirable in consequence of the numerous errors to which they have led. The fibrous tissue forms the stroma in which the separate cysts are imbedded. These morbid growths are most frequent in the generative organs; either in the ovaries, or near them, in the testicle, and in the female breast.

M. Müller has seen three forms—the simple cystosarcoma, cystosarcoma proliferum, and cystosarcoma with foliated warty excrescences from its cysts. In cystosarcoma simplex, the cysts contained in the fibrous sarcomatous texture have each their distinct membrane, the inner wall of which is simple, smooth, or at most beset with a few vascular nodules. To this class belong several cases described by Sir A. Cooper as hydatid tumors of the female breast, and likewise some of the preparations in Guy's Hospital, called "cystic tumor of the female breast."

In the second form, the sarcomatous mass is the same, but the cysts

* Illustrations of the Diseases of the Breast. London, 1829. p. 23.

within it contain younger cysts in their interior, which are attached to their walls by pedicles. An instance of this is afforded by the case of Mrs. King, described by Sir A. Cooper. This form of morbid growth is a repetition of the cystis prolifera, but imbedded in a sarcomatous mass, which constitutes the chief part of the tumor; and it may, therefore, be termed with propriety cystosarcoma proliferum. The pedunculated offsets from the cysts are hollow, and Sir A. Cooper saw some of them loose in the interior of the older cysts. It would likewise appear from an observation of Sir A. Cooper that the young cysts contain cholesteatomatous matter, for he found their interior composed of several laminæ, easily separable from each other, and presenting a glistening appearance.

The pedunculated bodies may, however, be sarcomatous.

"The third form, cytosarcoma phyllodes, differs greatly from the other two. The tumor forms a large firm mass, with a more or less uneven surface. The fibrous substance which constitutes the greater part of it is of a greyish white colour, extremely hard, and as firm as fibro-cartilage. Large portions of the tumor are made up entirely of this mass, but in some parts are cavities or clefts not lined with a distinct membrane. These cavities contain but little fluid; for either their parietes, which are hard like fibro-cartilage, and finely polished, lie in close apposition with each other, or a number of firm, irregular laminæ sprout from the mass, and form the walls of the fissures; or excrescences of a foliated or wartlike form sprout from the bottom of the cavities and fill up their interior. These excrescences are perfectly smooth on their surface, and never contain cysts or cells. Ths laminæ lie very irregularly, and project into the cavities and fissures like the folds of the psalterium in the interior of the third stomach of ruminant animals. In one instance the author saw these laminæ here and there regularly notched or crenated like a cock's comb. Sometimes the laminæ are but small, and the warty excrescences from the cysts very large, while in other instances both are greatly developed. Occasionally these warty excrescences are broad, sessile, and much indented; others have a more slender base, and somewhat resemble cauliflower condylomata. They are always formed of the same firm apparently fibro-cartilaginous matter as the non-hydatid portions of the tumor. The preceding description has been made from a cystosarcoma phyllodes of the female breast, weighing 2⅗ pounds, which was extirpated by M. v. Graefe, and is now preserved in the museum at Berlin, and is numbered 8906 in the catalogue.

On a microscopic examination of the firm mass, it is seen to have an indistinctly fibrous structure, but to contain neither cells nor cartilage corpuscules. It is very difficult to distinguish the fibrous texture of the mass; and this circumstance as well as the difference in the results of chemical analysis, suffice to distinguish the solid mass of cystosarcoma phyllodes from the tumor fibrosus s. desmoides. When boiled for twenty hours it yields no gelatine, and consequently consists of an albuminous substance; but the author never met with any other albuminous body of so great firmness. Whatever may be dissolved by long boiling is precipitated by corrosive sublimate, acetate of lead, tannin, and alcohol, but neither by acetic acid nor by alum. Once the author saw a large mass of cholesteatoma contained free within the cyst of a cystosarcoma phyllodes, which had been successfully extirpated by M. v. Brunn, of Coethen, and which was preserved in Meckel's museum at Halle. Tumors of this kind attain an enormous size; hitherto the author has seen them only in the female breast, nor are they even there of frequent occurrence. They are decidedly innocent, occur earlier than it is usual for cancer of the mamma to develop itself, and sometimes they appear even in youth; they have but little tendency to grow to the skin or to the subjacent muscles, and are not attended with retraction of the nipple. They are not disposed to soften internally, but continue to grow slowly until they have attained an enormous size, when they at length burst, and a very ill-looking suppurating fungus forms upon their surface. Even in this state, however, the operation has been performed with a successful result." 174.

These tumors have been described under the names of steatoma mammæ, carcinoma mammæ hydatides, carcinoma phyllodes, &c. and M. Müller appends some historical notices of the affection.

He adds a short section on the curability of the disease by operation. M. Müller remarks:—There are many and sufficient observations on record, all of which concur in representing cystosarcoma as curable by extirpation, and as having nothing in common with cancer, notwithstanding the extraordinary appearance which its warty excrescences or its laminæ exhibit. The case described by Chelius was cured by the operation. The same result followed the operation in the case from which the specimen in St. Bartholomew's Hospital was obtained; and amputation cured all the cases of hydatid tumors of the breast described by Sir A. Cooper. Swelling of the axillary glands is not a common occurrence, and, when it is met with, is the consequence of simple irritation, and subsides after the operation. The extraordinary forms which cystosarcoma phyllodes assumes, at once suggest the notion of its cancerous nature; and yet the disease is perfectly innocent, and as far removed from carcinoma as are those non-suppurating cauliflower condylomata of the penis, and of the female genitals, which have so often been mistaken for cancerous structures. The author has been fortunate enough to learn the history of a case which came under his own observation; and, likewise, of two others, the preparations from which are contained in the anatomical museum at Halle. In all of these the operation was completely successful. M. Müller adds:

"Although it is a well established fact that these forms of cystosarcoma are innocent, yet it must not be imagined that the appearance of cysts in a parenchymatous tumor necessarily excludes carcinoma, or a malignant nature. Indeed, just as cysts may be developed in every part of the organism, so it is quite possible that they may be formed in a carcinomatous structure. Among the many cases of innocent hydated tumors of the breast, Sir A. Cooper mentions one which was malignant, namely, a combination of cancer and hydatids. Moreover, the occurrence of carcinoma alveolare in the breast, although rare, has been observed by the author, and may lead to mistakes. In a carcinoma reticulare, the author saw part of the tumor presenting the ordinary structure of carcinoma alveolare, and containing cells filled with jelly. These cells might very easily have been mistaken for ordinary hydatids.

Carcinoma of the breast never attains that enormous size which cystosarcoma of the female breast sometimes reaches, before it passes into the open state. In Chelius' case the tumor had existed for ten years before the operation was performed, and a cystosarcoma proliferum had lasted for fourteen years before it was extirpated by Sir A. Cooper. The development of the tumor at a period of life when cancer mammæ is infrequent, its tardy progress, the enormous size which it attains without causing pain or producing, at the most, only very slight discomfort, and the fluctuation more or less distinct at the part where some cyst is situated, are points which, even before an operation, may lead to a tolerably certain diagnosis of these growths.

Enchondroma and cystosarcoma are the only forms of benignant tumor of the mammary gland which have hitherto come under our notice. The female breast is liable to innocent tumors of other kinds, which will hereafter claim our attention; as the albuminous and gelatinous sarcoma, and the fibrous tumor which in the breast, as well as in the uterus, is susceptible of transformation into bone." 182.

This completes our account of the part before us. We have given it in full and in detail, as we were anxious to place before our readers the opinions and observations of so accurate a pathologist as well as physiologist, on so difficult a subject. We shall return to it when the next part appears, and in the mean time we will remark that the work should be in the possession of every physician and surgeon who affects to practise with dignity and an expectation of success.

---

II. The length to which we have gone in pursuing the investigations of M. Müller into the nature and properties of carcinoma, must prevent us from entering so fully on the examination of the article "Cancer," in the Cyclopædia of Practical Surgery, as its merits could justify, and we should otherwise have done. The author, Mr. Spencer Wells, has bestowed on it great pains, and no less discrimination; and we think it offers a very favourable sample not only of his labours, but of the Cyclopædia itself.

We cannot pass it by with merely a barren encomium. There are many parts of it from which we may extract particulars necessary to complete the view of which M. Müller's work has offered us so large a portion. Over these parts we shall run, and if our notice of them is unconnected it is for the reason we have stated.

*Nosological Position of Cancer.*

Mr. Wells insists on the propriety of employing cancer or carcinoma as the generic term, scirrhus and encephaloid formation being only species. To these he adds, in accordance with the researches of Laennec, Otto, Cruveilhier, and Müller, "colloid" growths.

"The union of these morbid structures into a distinct class is not a mere nosological artifice: it is manifest that the formations, to which we propose to apply the generic term cancer, possess characters entitling them to be grouped together and separated from all others to which the frame is exposed. They agree *anatomically*, for they are all composed of a containing and a contained part forming a combination without its counterpart in the natural structures; they agree *chemically*, for they are all distinguished by the vast predominance of albumen in their composition: they agree *physiologically*, for they all possess in themselves the power of growth and of extension by continuity of tissue, that is, of assimulating to their proper substance the most heterogeneous materials an inherent tendency to destruction and the power of local reproduction; they agree *pathologically*, for they all tend to affect simultaneously or consecutively various organs in the body, and produce that depraved state of the constitution known as the cancerous cachexia. Their title to be united is quite as strong in respect of practical medicine and surgery, as in respect of scientific pathology, a consideration of the very highest importance. As respects the name to be given to the genus, cancer or carcinoma is clearly the best; to limit these terms to one particular tissue, when others possess the very properties on account of which they were originally employed, is a palpable contradiction."

To this no rational objection can be urged. It is the expression of the present advanced state of opinion. The following table seems to us a very good and useful one. It will assist the pathological student, and relieve him of the embarrassment which an over loaded nomenclature has given rise to. As Mr. Wells remarks, it will be seen from this table that cancer is synonymous *anatomice* with adventitious heterologous tissue. The fact of its being a tissue, that is, of its possessing structure, separates it unequivocally as a morbid product from others belonging to the same class, for example, pus and tubercle. The heterologous material of all carcinomatous formations is organizable, susceptible of vascular development, and hence of undergoing all the changes of increase and decay consequent on such susceptibility.

| Family. | Class. | Order. | Genus. | Species. | Varieties. | Synonyms of the Species. |
|---|---|---|---|---|---|---|
| Adventitious Formations. | Heterologous formations. | Tissues. | Cancer or Carcinoma. | Encephaloid. | Common vascular sarcoma, Mammary sarcoma? Abernethy.<br>Solanoid. *Recamier, Zang.*<br>Nephroid. Idem.<br>Napiform. Idem.<br>Carcinoma fasciculatum vel hyalinum. *Mueller.*<br>Fungus Hæmatodes. *Hey.*<br>Hæmatode Cancer. *Auct. Gall.* | Spongy or ossivorous tumour. *Ruysch. Palletta.*<br>Struma fungosa (testis). *Callisen.*<br>Spongoid inflammation. *Burns.*<br>Milt-like tumor. *Munro.*<br>Medullary Sarcoma. *Abernethy.*<br>Cerebriform disease or cancer. *Laennec*<br>Pulpy testicle. *Baillie.*<br>Carcinus spongiosus. *Good.*<br>Carcinoma spongiosum. *Young.*<br>Fungoid disease. *A. Cooper, Hodgkin.*<br>Medullary fungus. *Maunoir, Chelius.*<br>Acute fungous tumor. *C. Bell.*<br>Medullary cancer. *Travers.*<br>Cephaloma. *Hooper, Carswell.*<br>Carcinoma medullare. *Mueller.*<br>Soft cancer. *Auct. Var.* |
| | | | | Scirrhus. | Pancreatic sarcoma? *Abernethy.*<br>Napiform, Chondroid, *Recamier.*<br>Lardaceous tissue. *Auct. Gall.*<br>Carcinoma Reticulare. *Mueller.* | Carcinomatous sarcoma. *Abernethy.*<br>Carcinoma scirrhosum. *Young.*<br>Scirrhous cancer. *Travers.*<br>Scirrhoma. *Carswell.*<br>Carcinoma simplex vel fibrosum. *Mueller*<br>Stone cancer. *Auct. Var.* |
| | | | | Colloid. | Pultaceous cancer. Pearly alveolar ditto. *Cruveilhier.* | Areolar gelatiniform cancer. *Cruveilhier.*<br>Carcinoma alveolare. *Mueller.*<br>Gum cancer. *Hodgkin.* |

The heterologous character of carcinoma distinguishes it too from the analogous adventitious growths, as fatty, fibrous, and cartilaginous tumors. Mr. Wells, however, uses the terms analogous and heterologous in reference to external, not to microscopical characters.

Mr. Wells divides his article into two parts; devoting the first to the subject of cancer in general, in the second describing the disease as it occurs in those tissues and organs in which it is likely to come under the notice of the practical surgeon.

We pass by the *anatomy* of encephaloid disease, scirrhus, and colloid, and stop at their *physiology.*

In treating of the *Physiology* of cancer, Mr. Wells judiciously passes over, or very lightly alludes to, many of the ridiculous theories promulgated at various times. He examines more particularly the hypotheses of Hodgkin, Cruveilhier, Carswell, and Müller.

On that of Dr. Hodgkin we need not speak, as it has been sufficiently examined and objected to. Our readers, perhaps, are less familiar with the opinions of Cruveilhier, and we shall therefore notice them.

"M. Cruveilhier's doctrine embraces two essentially distinct propositions. First, he regards all heterologous formations, 'as the exclusive result of a successive deposition of morbid products in the cellular element of organs;' he believes that 'this cellular element is alone affected; that the proper tissue of organs is in itself incapable of undergoing any organic lesion, except hypertrophy and atrophy; that at first rendered hypertrophous by the state of irritation existing in the neighbouring cellular membrane, these proper tissues subsequently become atrophous, and finally disappear in consequence of the pressure they undergo from the diseased formation.' But this unalterableness of composition of the organic tissues is the least important part of the doctrine; for, secondly, 'it results from his researches that the formation of cancer, like all nutritive phenomena, physiological and morbid, takes place in the venous capillary system; that from it the morbid products are poured into the cellular membrane, either by exhalation or through lacerated openings.' This latter proposition, as it affects cancer, is evidently only a part of his more general theory, according to which the various phenomena of nutrition, secretion, and inflammation, are accomplished, not as has heretofore been believed in the arterial, but in the venous capillaries."

Mr. Wells remarks that, much as M. Cruveilhier insists upon the doctrine that carcinoma occurs primitively and invariably in the seat he has assigned to it, yet the only fact that he advances in support of it is the occurrence of carcinomatous matter in the veins.

From the full account which we gave, at the time of their publication, of Dr. Carswell's Fasciculi of Morbid Anatomy, our readers may perhaps remember his opinion—*that,* carcinoma originates in three positions—in the molecular structure of organs as a product of nutrition; on free serous surfaces, as a result of secretion; and in the blood. It is in the latter, indeed, that Dr. Carswell places the real site of cancer, and the hypothesis is one of the most important description. Mr. Wells, therefore, collects what is known upon the subject.

The earliest careful observations, he says, respecting the presence of cancerous matter in the vascular system appear to have been by M. Velpeau. In 1824 this inquirer read an essay to the Academy of Medicine, containing two cases related with much precision. In one of these an encephaloid mass appeared in the vena cava communicating with similar structure in the kidney; and an enormous coagulum extending from the iliac veins through the cava inferior to the right auricle, presented from place to place the characters of encephaloid matter of varying firmness. The coagula in the heart had apparently formed after death, yet in one of them, placed between the walls of the ventricle and the tricuspid valve, was contained "some substance of purulent *or* encephaloid aspect, like that contained in the kidney." In 1829 M. Andral announced his having discovered carcinomatous matter in the trunk and minute ramifications of the pulmonary artery; in other instances, in the branches of the vena porta, &c. In the same year M. Cruveilhier, in describing a case of cancer of the uterus and vagina, stated that on cutting the latter membrane slantingly, he ascertained, with the aid of a lens, that the venous areolæ of the vaginal mucous membrane were crammed with encephaloid easily expressible from their interior.

These facts all prove the presence of carcinoma in the interior of the venous system. The different observers differed much in their conclusions from these facts. But Dr. Carswell goes farther than any.

"He affirms not only that the morbid production may be discovered in vessels having no direct communication with an organ affected with the same disease (as when confined to a small extent of the vena porta), but that there are cases in which the venous blood alone is found to be the seat of the morbid accumulation. Of the first of these observations we readily admit the accuracy, but it by no means disproves the fact of venous absorption. There does not seem to be any direct communication between the parenchyma of the lungs and the bones of the extremities; yet the celebrated experiments of M. Cruveilhier show that a single globule of mercury introduced into the medullary cavity of the femur may be detected weeks after in the pulmonary tissue. Nay more, they have proved that mercury, introduced into any part either of the general or portal venous systems, may reach in the first instance the capillaries of the liver, in the second those of the lung. With respect to the second observation, we can only say, that among a vast number of post-mortem examinations, conducted by men familiarized with such researches, we have never seen a case of the description referred to, we have never heard of one, and we have in vain searched periodical works for details demonstrating the reality of its occurrence. Were the fact substantiated it would furnish strong evidence of a *primary* cancerous alteration of the blood, provided the adjoining venous structure were shown to be wholly unimplicated; but while unsupported by a full narrative of the cases from which it professes to derive the doctrine of such alteration, no matter how eminent the individual by whom it is adopted, can only, we apprehend, be regarded in the light of an hypothesis. The fact that the heterologous substance is not discovered in the arterial system (Cruveilhier's statement [p. 594] requires substantiation) is accounted for by Dr. Carswell by supposing that the contractile power of the arteries prevents stagnation of their contents: how far this may be considered an answer to an obvious argument against his theory, we are unprepared to say; it is certain that it is far from convincing.

Further, as Dr. Carswell regards the deposition of carcinoma, through the processes of nutrition and secretion, as the resalt of its *separation* from the blood, cancerous vitiation of that fluid, preceding the origin and co-existing with the growth of the local malady, is a *sine quâ non* of the development of the latter: according to him the blood is, in a word, the sole primary seat of the disease. Hence the material element of the affection is in circulation in cases, for instance, of scirrhus of the mamma, which remains almost stationary for years, is unattended with secondary formations in other parts of the frame, and gives rise to no marked constitutional disturbance. Now this idea is by others rejected as inadmissible; can we readily comprehend, they allege, how blood holding cancer in suspension can furnish the materials of normal secretions and perform its functions in such manner as to cause no perceptible deviation from the standard of health? How comes it again, it is urged, if this doctrine be well founded, that after the removal of a cancerous tumour the process of cicatrization frequently goes through its stages with regularity, though when the patient dies, one or more viscera are found affected with the disease in such manner as to leave little doubt, in some cases none, that the morbid growths discovered in them were in existence before the performance of the operation. If these facts are reconcileable with the theory espoused by Dr. Carswell, he has at least failed to make manifest their compatability therewith. For our own part, while we distinctly avow that the difficulty of explaining the apparent contradictions involved in this doctrine furnishes no *proof* of its unsoundness, we regret it as unestablished by experience.

Again, though we look upon it as almost demonstrated that cancerous matter found in the veins has in the majority of cases come there by absorption (see the section on Pathology), yet we are by no means desirous of upholding an exclusive opinion on the matter. Facts well observed prove, in the first place, that growths external to those vessels, in some instances perforate their coats, protrude into, and extend laterally in, their interior. Nor do we deny the possible conversion of coagulated blood into encephaloid; we simply alledge that such transformation is as yet unproved. Thirdly, published cases tend to prove the occasional production of carcinoma by the coats of the veins; circumscribed tumors are sometimes found attached to the walls of these vessels by a simple or multiple pedicle (not unfrequently dilating the vein, as M. Cruveilhier observes, into an ovoid ampulla simulating a cyst). These tumors are evidently in organized connexion with the vascular tunics—a fact arguing in its favour, though it by no means demonstrates their production thereby."

Mr. Wells observes that it may fairly be inferred that the primary seat of carcinoma is the intervascular interstices of all the organized tissues and parenchymata, in rare instances possibly free serous surfaces and the interior of the veins. Wherever there are capillary vessels composing a nutritive or secretive apparatus, cancerous matter may be produced; that it may be sometimes retained in these may reasonably be supposed, but there is at present no proof of the fact. Secondly, the proximate cause of the formation consists in a perversion of the facts of nutrition or secretion. The state of the blood is a difficult problem. Possibly, at the earliest period, it may not preserve its perfect normal constitution; and probably, when secondary cancerous formations occur, its state must exercise a material influence in their production or their growth.

A very full account is given of the *growth* of carcinoma. Our readers are aware that Müller contends that the microscopical elements of cancerous growths and their mode of propagation are identical with those not only of benignant tumors, but of the normal embryonic tissues. He therefore infers that carcinoma is not a heterologous formation. And, in the sense in which the term *was* received, there cannot be a doubt, that, presuming his premises to be correct, he is right. But Mr. Wells remarks that such identity simply shews that the heterologous character, visible and palpable as it is, is produced not by the nature, but by the mode of combination and arrangement of the ultimate physical elements of the diseased growth. As well might it be contended that calomel and corrosive sublimate are not heterologous to each other, because they are both composed of atoms of chlorine and mercury. Our readers will perceive that the question is merely one of words. It is only necessary to define what heterologous shall signify to settle it.

*Effects of Cancer on Adjoining Parts.*

A complete account is given of these, and we feel disposed to notice it. These effects are by no means uniform.

*a.* In some situations cancerous deposits may acquire considerable volume without apparently influencing the substance of the part in which they are formed. This is observed in the lung and in the encephalon, even when no cyst intervenes between the abnormal and natural tissues; hundreds of tumors are sometimes found in the liver, separated by perfectly normal structure.

*b.* A very simple effect is *condensation* of the surrounding substance. This is familiarly seen in the condensation of the adjoining cellular substance by subcutaneous tumors. Aided in some instances by slight hypertrophy of the cellular laminæ, a pseudo-cyst investing the morbid growth is thus developed. The formation of this natural barrier between the diseased and healthy tissues, more frequently observed in the case of encephaloid than of scirrhus, no doubt helps to defend the latter from infiltration with the morbid matter. The substance thus cut off, as it were, from surrounding parts, has less tendency to contract adhesions with them.

*c.* These tumors produce *mechanical effects* regulated by their size and position. Thus if developed around the vena porta, they interfere with the circulation in that vessel and induce ascites, &c.

*d. Discoloration* of the neighbouring parts is sometimes effected in a manner as yet unexplained: thus the fat adjoining mammary carcinoma assumes a yellowish saffron tint. In advanced stages of cancer in the same organ, the integuments acquire a dingy green or olive hue, ascribable, as Dr. Hodgkin mentions, to changes in the venous blood.

*e.* Cancer, when so situated as to oppose the onward movement of the contents of hollow viscera, leads to *hypertrophy* of the muscular apparatus behind the obstruction. Thus in anal carcinoma the muscular tunic of the rectum undergoes thickening.

This is a mere consequence of the increased call on muscular exertion.

*f.* Another change of nutrition occurs. In scirrhus of the mamma, as has been observed by Sir C. Bell, Scarpa and others, the proper tissue of the gland occasionally suffers *atrophy;* in such manner that a breast, in which an heterologous growth is encased, hardly or not at all exceeds its healthy fellow in bulk. In other cases, on the contrary, the diseased organ undergoes general enlargement, or parts of it may be atrophous and others tumefied. The first of these cases is termed by Recamier *hypertrophous,* the second *atrophous engorgement:* the combination of the two states produces inequalities and bumps.

*g.* Carcinoma sometimes produces *absorption* of the part on which it presses. This is exemplified in the progress of meningeal cancerous growths which, after producing perforation of the cranium, sprout out through the opening.

*h. Serous infiltration* is frequently observed round subcutaneous and glandular tumors; and it is stated by Cruveilhier, that in cases of cancer of the cylindrical bones, the medulla is not unfrequently saturated with serosity.

*i. Inflammation* almost invariably occurs sooner or later in the tissues investing carcinoma: it is either eliminatory or not in its effects. As instances of the latter may be cited, gelatiniform softening sometimes produced in the cerebral tissue subjacent to meningeal cancer; peritonitis spreading from the seat of superficial carcinoma of the liver and giving rise to ascites, &c.

*j. Fibrous transformation* is sometimes observed. Cruveilhier has found the hepatic tissue between cancerous tumors converted into true fibrous substance, and relates a case of mammary carcinoma, in which the pectoralis muscle had undergone a similar change of structure.

*k.* The extension of the disease to contiguous textures by *infiltration* occurs in the case of the three species, but not with the same frequency. Encephaloid has been by some considered more prone to push the adjacent parts aside, than to convert them into matter like itself: Abernethy considered this a fact of sufficiently constant occurrence and importance to warrant a complete separation of scirrhus and medullary sarcoma. But it is far from unusual for encephaloid of the cellular membrane of the limbs to spread to the adjoining tissues. Cerebriform growths originating under the peritoneum spread to the bones of the pelvis, and even of the thigh: this progressive destruction even of the hardest tissues was by Ruysch esteemed so important that he named the disease ossivorous tumor. On this point indeed, there is much difference of opinion amongst authors. But there can be no question that encephaloid, though it does invade contiguous tissues, is not so prone to do so as scirrhus. The extension of scirrhus to contiguous textures is a very constant phenomenon; as it spreads, the scirrhous growth loses its circumscribed, rounded, moveable character. Perforation of the hollow viscera consequent on conversion of their walls into cancerous matter is an occurrence familiar to the clinical observer. Such destruction of the recto and vesico-vaginal septa forms one of the most deplorable epiphenomena of uterine cancer.

*l.* Mr, Wells passes in review the various modes or degrees in which the different tissues are involved during the spread of cancer. The *albugineous* tissues generally are remarkable for their power of resisting cancerous infiltration: a fact illustrated by the effect of fasciæ on these growths. The *arterial tissue* in connexion with carcinoma commonly remains unbroken for a length of time, while the investing textures undergo rapid destruction. But that the arteries are not always spared, appears from their occasionally giving way with dangerous facility, when ligatured in the immediate neighbourhood of cancerous formations. Hematemesis has followed erosion of the splenic or coronary arteries in cancer of the stomach, &c. The adjoining arteries are sometimes dilated; Cruveilhier has seen nameless ramusculi running to a mammary cancer acquire the caliber of the radial. The *veins* in communication with these products undergo singular enlargement; but a similar effect is produced by other tumors. Numerous observers have ascertained that these vessels become morbidly friable, a fact particularly insisted on by Recamier. And cancerous matter may be found in their interior. The condition of the adjoining *nerves* varies. Their tissue may retain all its normal characteristics; it may be absorbed from pressure; become cancerous; or the nervous substance may alone be absorbed, while the neurilemma, thickened and indurated, assumes the characters of fibrous tissue. They have been more commonly observed to undergo encephaloid than scirrhous infiltration. Breschet has, however, found the areolæ of their tissue infiltrated with a liquiform matter resembling that of scirrhus. The *excretory ducts* of glands are sometimes stuffed with the morbid matter: the ureter has thus been found partially or wholly obstructed.

The *Pathology* of carcinoma is treated at some length, and with ability. Mr. Wells first adverts to the greater frequency of the disease in some organs than in others. After noticing the various hypotheses upon the subject, hypotheses that flatly contradict each other, he concludes very fairly, that we know nothing of the matter.

Then Mr. Wells pursues the *Anatomical course* of the disease. After observing that it may be limited to one organ throughout, he remarks that it may spread to a multitude of parts, which are then said to be affected with *secondary cancer*.

The mechanism of transmission probably varies as the parts contaminated are in proximity or remote. In the former case the propagation is effected by the progressive interstitial deposition or infiltration of the morbid matter in tissues naturally continuous or contiguous; or parts simply placed in juxtaposition or approximation may, according to Dr. Hodgkin, without the pre-establishment of adhesion, contaminate each other. As instances of the latter mode of contamination, cases are referred to, in which an ovary, the mesentery, or the liver being the seat of a malignant tumor, the parts in contact with it, whether convolutions of intestine or abdominal parietes, become similarly affected.

The implication of remote parts seems only possible through the medium of the lymphatic or the venous systems.

"The condition of the lymphatic structure in communication with a cancerous tumor varies. In some cases it is, to all appearance, normal; in the vast majority of instances, when the disease has existed for any length of time, the glands are affected with simple or cancerous induration, while the vessels leading to them are either filled with the heterologous matter, or, as is more common, no modification is discernible in their anatomical state. Now when the tubes are themselves loaded with cancerous substance, and are, for example, traceable so loaded from the diseased organ even to the thoracic duct, without any evidence existing of the matter being a product from the walls of the tubes themselves, the implication of the lymphatic system is evidently the result of absorption. But when the glands are cancerous and the connecting tubes in the natural state, the condition of the former is not thus so satisfactorily explicable. The difficulty may, it is alleged, be obviated by supposing the secondary formation induced by congestion of the glands, itself excited by the irritation attending the progress of the primary growth. We are at a loss to understand why the vessels themselves, in closest proximity to the disease, should not suffer, were this conjecture well-founded: the analogy of common or venereal irritation will not fairly apply here. Maunoir observes that in the advanced stage of encephaloid, lymphatic glands unconnected with the tumor by vessels sometimes become affected, and Sir A. Cooper has seen the axillary glands of the opposite side diseased in cancer of the mamma. In these cases the lymphatic carcinoma must be admitted to occur independently of the existing growths, unless the statement of Scarpa, that the cancerous ichor sometimes passes circuitously along the anastomosing lymphatic vessels be well founded."

We confess that this seems to us straining at gnats. It is easy enough to conceive the arrest of carcinomatous matter, and the induction of carcinomatous action in the intricate tissue of an adenöid gland, when the open channel and simple texture of the inferent or of the efferent vessels have favoured their escape. It is, we think, going out of the way *for* a difficulty, to reject direct contamination, when glands in direct communication with a cancerous formation are affected with it.

"As it is necessary to admit that the process of absorption, as a part of nutrition, is constantly going on in cancerous growths, it may be inquired why distant contamination does not occur from their earliest deposition, A parallel case may be cited. The pyogenic membrane or enclosing cyst of abscesses is by all admitted to be an absorbing and secreting agent, yet in how few cases of abscess does pus find its way into the circulation. Both facts may be explained by supposing that these vessels normally exercise a decomposing action on the purulent or cancerous matter, which they cease to do on some occasions, admitting it then into their interior with its natural properties."

It is obvious that *amount* as well as *nature* of absorption may vary under different circumstances, and the quantum of morbid matter necessary to produce secondary contamination is what we have no means of measuring.

" But the occurrence of secondary carcinoma in localities free from lymphatic communication with the seat of a primary growth, remains to be explained. In these cases it appears hardly possible to doubt that the venous system is an agent of translation: while M. Cruveilhier affirms that such is the case with all the earnestness of conviction, Müller simply admits the possibility of the absorption and diffusion of the germinal nuclei giving rise to secondary formations. If it be true that we still want the material demonstration of this—if no investigator has yet discovered the cancerous matter actually *in transitu* with the blood in the veins leading from one diseased organ to another—still the following arguments may be adduced as affording strong presumptive evidence in favour of the reality of such transmission. 1. Cancerous matter exists in a multitude of cases in the veins of the diseased part; now this is obviously a most favourable circumstance for its circulation with the returning blood. 2. The rapidity of the successive development of the disease in different organs, sometimes observed, seems only producible by the agency of a fluid which, like the blood, pervades them all. 3. The liver and lung, the two organs in which foreign bodies introduced into the circulation are almost invariably observed to stagnate, are by far the most frequent seat of the secondary development of carcinoma. 4. Secondary cancer in the liver and lung occupies the same elementary seat as pus formed in those organs consecutively to the existence of suppuration in a distant part of the frame (metastatic abscesses). The granules of the former organ are in both instances the element affected. Dr. Carswell, arguing for the primary vitiation of the blood, affirms that the fact of absorption is disproved by minute anatomical details; but gives us no information as to the presumed nature of these. Dr. Hodgkin is persuaded that the consistence of the matter is sufficient evidence 'of its not having been transported along the vessels in which it is found,' (of course this applies *à fortiori* to secondary tumors in the parenchymata) and believes its existence in the veins and lymphatics to be the result of 'inflammation of the malignant growth and the natural structures connected with it.' The fact of inflammation occurring under these circumstances is unestablished; we shall presently recur to the objection founded on the consistence of secondary formations. According to Müller the presence of cancer in the venous capillaries 'appears simply to indicate inflammation of those vessels:' a notion discordant with the well-established fact, that the morbid matter is found in perfectly healthy portions of the venous system.

Nevertheless some apparently serious objections may be raised to the doctrine of venous transmission. If such, it may be urged, be the mechanism of the production of carcinoma in distant organs, why is such production not a constant phenomenon? why should cancerous disease ever terminate the existence of individuals without having manifested itself *materially* in more than a single spot? A satisfactory answer cannot in the present state of the science be easily found. It must, however, be recollected, that the presence of the morbid substance in the veins has not been proved to be a constant condition of its existence in every form of the disease; secondly, that when the minute veins are blocked up, the circulation in the portions of tube immediately in front of the obstruction can only go forward by anastomotic communications; and that, thirdly, the morbid substance is in many instances completely cut off from the general circulation by sanguineous concretions. Of this last fact published engravings of carcinoma furnish examples. Another objection to this doctrine may appear in the multitude and large size of secondary formations; it is physically impossible, it may be alleged, that these should have been the produce of absorption from a mass, itself smaller than many among them, and which at no period of its growth suffered apparent diminution of bulk. But it is not meant in the remotest degree (and this is the answer to those who object to the origin of secondary carcinoma in absorption on the score of its consistence) that consecutive formations are composed of the actual substance of the primary mass, though even this does not seem, from the evidence of infinitely rare cases, to be impossible; the micrography of cancer shows that the translation and deposition of a few cells only from the original nidus might lead to the development of the largest mass; each cell is in itself *the possible embryo of a tumor.* There is yet another fact apparently subversive of the notion of venous transmission. The detritus and ichor of carcinoma have been injected into the veins of animals, but no development of cancerous structure has resulted from the experiment. Granted, but this simply proves—and it does so perhaps as conclusively as any other adducible argument—the absolute necessity of predisposition for the production of the disease; without this even its material constituent manifests itself only as an irritative agent."

On the whole, it appears to us, that, as in the case of secondary abscesses, the arguments as well as the facts in favour of venous transmission, preponderate; and if a cachexia has led to the original formation of the carcinoma, that cachexia must probably be greatly aggravated by influx into the system of carcinomatous particles through the lymphatics and the veins.

Mr. Wells observes that the *condition* of the solids and fluids of subjects dying with cancerous disease has scarcely been investigated. Lymphatic and visceral secondary contamination are the only lesions recognised as attendants on the progress of disease.

"M. Louis found that the mean volume of the heart was less in subjects dying from cancer, especially of the stomach and uterus, than in those succumbing under any other disease; and that while the aorta measures 34½ lines (Fr.) in width at the edge of the sigmoid valves in subjects dying of acute diseases between the ages of 30 and 40, and 32 in the victims of phthisis, it only measures 30 in carcinomatous subjects of the same age.

Tubercle and cancer rarely co-exist. In fifty-two autopsies of cancerous subjects collected from various sources we found but three examples of the anatomical characters of phthisis. The difference of the ages at which the two diseases are most prevalent would lead us to expect a result of this kind, independently of any influence which the formation of one may have in excluding that of the other form of morbid matter. It is worthy of remark, that in the three instances of co-existence referred to, the cancerous affection was of the scirrhous species, and the mean age of the subjects thirty-seven. The absence of tuberculous diseases in all the cases of encephaloid (thirty-one in number) argues strongly against the opinion of those who either consider the latter allied to scrofula, or with Mr. Travers actually regard it as cancer modified by a strumous constitution: indeed we have never yet met with a solid argument in support of this doctrine."

We think Mr. Wells rather positive in this instance. The analogy, such as it is, between encephaloid and scrofula, has always seemed to us mainly to consist in this—*that* encephaloid, unlike scirrhus, affects the young—and *that*, it is very often witnessed in persons of a scrofulous appearance. These are not very conclusive arguments, it is true.

Mr. Wells has devoted considerable attention to the *etiology* of cancer, and the details which he has entered into are both instructive and interesting. Indeed, this may be regarded as the best part of the article.

*a. Contagiousness.*—Mr. Wells remarks, that it has, till very lately, been supposed that cancer was inoculable, or even infectious. "The grounds for the belief consisted on the one hand of a few ill-authenticated statements in the older writers respecting the development of the disease, in persons presumed to have been previously healthy, as a result of close and habitual contact with cancerous subjects; on the other, of an experiment by Peyrilhe, in which that surgeon introduced some cancerous matter under the skin of a dog, and produced, as he implies, a carcinomatous ulcer. Admitting the authenticity of the first order of facts, they can only prove the fact of coincidence. As to the experiment of Peyrilhe, the only ascertained results were violent inflammation and gangrene; the ultimate effect was not known, as the animal was lost sight of.

The accurate observations of modern pathologists have settled this question in the negative. First, Alibert, Biett, and several pupils of the Hôpital St. Louis inoculated themselves with the ichorous discharge of cancerous ulcers without suffering any particular effect from the operation: Secondly, Dupuytren introduced pieces of cancerous tissue into the stomachs of animals, and injected the matter from ulcers into the veins and splanchnic cavities without producing any result except those caused by irritating substances generally: Thirdly, among a considerable number of men who had habitual intercourse with women affected with ulcerated cancer of the cervix (of which the existence was established by post-mortem examination) not one presented the slightest symptom of such disease (Bayle): Fourthly, surgeons have frequently wounded themselves in the extirpation and dissection of cancerous growths, without suffering in consequence from carcinomatous disease. (Jäger.)

We would observe, that it does not follow, because the disease is not communicated in certain experiments, that long continuance in the same air, and close communication with a patient labouring under cancer, would not predispose to that complaint. It has been observed by modern surgeons, as well as by old ones, that females who have tended on others labouring under cancer, have been so frequently affected as to raise a fair suspicion of something more than a coincidence. We suppose that Mr. Wells will hardly deny the frequency with which the wife of a phthisical husband, or husband of a phthisical wife, becomes the victim of phthisis. Yet we are not aware of any facts which prove the direct communication of phthisis by experiment, nor is it at all likely. We have lately seen a case which would seem to show the direct transmission of cancer. A gentleman had malignant ulceration of the penis and in the groins, and died of it. His wife was attacked with similar ulceration in the vagina. We would not, therefore, discard the notion of infection as altogether inadmissible.

*Age.*—The different species of carcinoma may orignate at every period of existence. In a remarkable case described by Billard, development of scirrhus had taken place in the heart during intra-uterine life; under the head of Meningeal Encephaloid we shall have occasion to refer to two cases in which that affection existed at birth; and Mr. Travers has figured a remarkable specimen of congenital encephaloid of the eye observed by himself and Sir A. Cooper when the infant was eight months old; at birth the eyeball was as large as a walnut. M. Cruveilhier has, on the other hand, known uterine cancer manifest itself at the advanced age of eighty-four. The general sense of authors on this point, however, is, that the disease rarely occurs in early life, seldom originates in old age, and is especially frequent in both sexes between the ages of thirty-five and fifty."

To test this, Mr. Wells has examined the returns of deaths supplied to the Registrar-General, under the late Registration Act. He has accordingly compiled a table from twelve hundred cases.

TABLE

*Shewing the absolute mortality from Carcinoma in both sexes and at all ages.*

| AGE. | MALES. | FEMALES. | BOTH SEXES. |
|---|---|---|---|
| 1 month | | | |
| 2 ,, | .. | 1 | 1 |
| 3 and under 6 | .. | 1 | 1 |
| 6 ,, 9 | | | |
| 9 ,, 12 | | | |
| Years. | | | |
| 1 ,, | 2 | 1 | 3 |
| 2 ,, | 1 | 4 | 5 |
| 3 ,, | .. | 1 | 1 |
| 4 ,, | .. | 1 | 1 |
| 5 and under 10 | 3 | 2 | 5 |
| 10 ,, 15 | 1 | 4 | 5 |
| 15 ,, 20 | 3 | 5 | 8 |
| 20 ,, 25 | 4 | 2 | 6 |
| 25 ,, 30 | 1 | 13 | 14 |
| 30 ,, 35 | 6 | 23 | 29 |
| 35 ,, 40 | 15 | 43 | 58 |
| 40 ,, 45 | 19 | 77 | 96 |
| 45 ,, 50 | 23 | 98 | 121 |
| 50 ,, 55 | 34 | 130 | 164 |
| 55 ,, 60 | 35 | 120 | 155 |
| 60 ,, 65 | 44 | 110 | 154 |
| 65 ,, 70 | 45 | 88 | 133 |
| 70 ,, 75 | 35 | 69 | 104 |
| 75 ,, 80 | 30 | 49 | 79 |
| 80 ,, 85 | 16 | 28 | 44 |
| 85 ,, 90 | 1 | 8 | 9 |
| 90 ,, 95 | 2 | 1 | 3 |
| 95 and upwards | 1 | .. | 1 |
| Totals.. | 321 | 879 | 1,200 |

And we subjoin another table, calculated on the preceding, corrected by the Population Estimates of Mr. Rickman.

TABLE

*Shewing the proportion of deaths from Cancer in* 1,000 *living of each sex at the different ages.*

| AGES. | MALES. | FEMALES. | MEAN. |
|---|---|---|---|
| Under 5 | ·006 | ·017 | ·012 |
| 5 and under 10 | ·007 | ·004 | ·006 |
| 10 " 15 | ·002 | ·010 | ·006 |
| 15 " 20 | ·009 | ·017 | ·013 |
| 20 " 30 | ·010 | ·024 | ·017 |
| 30 " 40 | ·058 | ·152 | ·105 |
| 40 " 50 | ·140 | ·983 | ·561 |
| 50 " 60 | ·290 | 1·066 | ·678 |
| 60 " 70 | ·636 | 1·192 | ·919 |
| 70 " 80 | ·935 | 1·421 | 1·178 |
| 80 " 100 | 1·207 | ·973 | 1·089 |
| All ages .... | ·103 | ·245 | ·174 |

Mr. Wells observes that this table demonstrates the inaccuracy of the commonly received opinion, that the tendency to cancer reaches its maximum between the thirty-fifth and fiftieth years. The mortality, on the contrary, goes on steadily increasing with each succeeding decade until the eightieth year. The mortality from the disease is lowest at the same period of life as the general mortality from all causes indiscriminately, namely, from the tenth to the fifteenth year. "The law of mortality," he goes on to remark, "differs strikingly in the two sexes; the most general character of this difference being the greater regularity of increase with advancing age among males. Passing over the first twenty years, at which period the number of cases is probably too small to admit of very useful comparison, we find, it is true, that the number of deaths from æt. 30 to 40 is in both sexes about six times greater than from æt. 20 to 30; but here the similitude of increase ceases: in the next decennial period the deaths among females augment more than sextuply, while the mortality of males only increases two and a half times, &c."

Mr. Wells comments on the enormous and abrupt increase of female mortality between the ages of thirty and fifty; an increase which accounts for the erroneous notion that the disease reaches its maximum frequency at that period of life, and lends support to the current belief respecting the connexion of uterine and mammary cancer with declining activity and cessation of the genital functions.

The different species of cancer are far from being equally common at all ages: scirrhus is essentially a disease of adults, while encephaloid is the form usually assumed by the morbid growth in young subjects; colloid appears so far to have been only observed in adult individuals.

"Again, the locality of carcinoma is manifestly somewhat under the influence of age. Previously to puberty, the disease is most common in the eye, the lymphatic system, the brain, and the cellular membrane of the extremities; the uterus, mamma, stomach, liver, intestines, and bones enjoy comparative immunity until the thirtieth year. Exceptions to this rule are no doubt met with; Dr. Carswell is of opinion, that the exceptional occurrence of the disease during infancy or early youth in the latter class of organs depends upon their premature or præternatural functional excitement, and the statement appears to hold good in respect of the uterus, testes, and ovaries."

*Sex.*—In the last half year of 1837, it appears that, of 1228 individuals who died of carcinoma during that period, 355 were males, 873 females. By calculating the rate of mortality in proportion to the numbers living of each sex, we learn that the annual mortality from the disease is ·103 per thousand in males, and ·245 in females: the excess on the side of the latter is therefore as nearly as possible as 2·5: 1. This difference is the more remarkable, from the fact that the mean rate of mortality from all diseases is 20·8 per thousand among males, while it is 19·7 among females.

The *influence of town or country habitation* is examined. Breschet asserted as an axiom that cancer is rare among persons employed in agriculture. What do Mr. Farr's tables say? From these, says Mr. Wells, we learn, that in the metropolis 185 deaths occurred from cancer, while five counties of nearly equal population, Cornwall, Dorsetshire, Devonshire, Somersetshire, and Wilts, furnished 126 deaths from the same disease. But the excess, on the part of London, is not even so marked as would on first sight appear; for, calculating from the estimated population in October 1837, the annual mortality is ·206 per thousand in the capital, ·143 per thousand in the counties. And, again, the number of deaths from this affection in a number of our principal manufacturing towns (Leeds, Liverpool, Manchester, Sheffield, &c.), with a total population of 1,762,710, exposed *per eminentiam* to what are esteemed the insalubrious conditions of the life of town artisans, is 152; while in a number of rural districts, containing 1,776,980 souls, 163 individuals fell victims to the disease. Finally, comparing the deaths from cancer in the metropolis and provincial towns on the one hand, with those in all the rural districts referred to on the other (337 in the former, and 289 in the latter), we obtain an annual mortality of ·189 per thousand in large towns, and of ·165 in the country. The character of this result is rendered more distinct by comparing it with the rate of mortality, from all diseases indiscriminately, in the same towns and counties: the annual mortality in the former is 27 per thousand; in the latter 16·9 per thousand, showing a remarkable minority in favour of a country life.

Thus a town life exercises no serious influence on the production or mortality of cancer, and when we allow for the circumstances that many patients with cancer in the country, remove to towns for advice or for the hospitals, we shall be the more confirmed in our opinion. When any excess occurs in town mortality, it is curious that it is due to the females.

Mr. Wells is unable to arrive at any satisfactory estimate of the influence of occupation or trade on the disease: nor is that of celibacy or matrimony very well made out, though the following is offered as an approximation to the truth:—of the unmarried ·07; of the married 0·48; of widows 1·32 per thousand die from carcinoma. But these proportions are probably mainly influenced by the mean *age* of the different classes of women furnishing them. Mr. Wells appears to doubt the agency of mental affliction, and to lean feebly on hereditary transmission. For our parts we attach more faith to both.

On the *causes* of cancer we do not see much to detain us. Mr. Wells is sceptical of the efficiency of local injury as an exciting cause, and, of course, it is greatly over-rated; yet, a predisposition to the disease existing, nothing is more likely than that local injury should help to light it up. And, however writers, like M. Bouillaud, may ridicule the notion of *predisposition*, the common sense of men rises superior to their verbal quibbles and scholastic subtleties.

Mr. Wells endeavours to apply statistics to the *frequency, duration*, and *influence of season* on carcinoma. But however laudable the attempt, we do not think that the time is come for any reliance to be placed on the results of arithmetic calculations. The only table we are disposed to notice is the following:

| | From Cancer. (two and a half years.) | From Phthisis. (two years.) |
|---|---|---|
| Proportion of deaths in every 1000 deaths. | ·671 | 196·563 |
| Proportion of deaths in every 1000 living. | ·174 | 3·963 |
| Absolute number constantly ill, supposing the diseases mortal and of the mean duration above stated. | 6·140 | 111·416 |

It appears that, as is the case with most chronic diseases, more persons with cancer die in the inclement than in the milder months.

We would draw attention to the following circumstance in connexion with the *symptoms*.

"The vascular character of encephaloid seems to render it an *à priori* probability that under favourable circumstances *pulsation* might be detected in it. The existence of pulsation is in fact one of the signs of meningeal encephaloid growths protruding through the cranium; but observers are not agreed as to whether this is a motion transmitted from the subjacent brain or arising in the substance of the tumor itself. The same indecision exists with respect to the beating noticed by Dr. W. Stokes in a case of intra-thoracic carcinoma. On the other hand Dupuytren makes no question of the occurrence of interstitial pulsation, and describes the phenomenon as at first deep-seated, becoming gradually more superficial and distinct, isochronous with the pulse, unattended with rustling sound (*bruissement*), accompanied sometimes in advanced cases with general expansion of the mass, ceasing when the chief artery leading to the part is compressed, and produced by simultaneous dilatation of all the minute arteries of the tumor."

What Mr. Wells speaks of so hesitatingly, occurs, for we have ourselves twice seen it. In one instance the tumor was in the groin and outer side of the thigh, in the other in the iliac fossa. In the former instance, the femoral artery was tied, under the supposition that the tumor was an aneurysm. And, if we remember aright, the same thing occurred to Mr. Guthrie.

Before we quit Mr. Wells, we may allude to the sense in which he uses the terms diathesis and cachexia. By *diathesis*, he understands the state of constitution in which the multiplication of cancer occurs in the economy—by *cachexia* the sum of general symptoms attending the disease. Mr. Wells observes, in reference to M. Cruveilhier, that, "considering the cancerous cachexia and diathesis as one and the same thing, he explains the occurrence of both on the principle of venous transmission :—hence the existence of either involves that of the other. In this confusion of diathesis and cachexia the Parisian pathologist is joined by Müller. Yet that the distinction between the two is not a scholastic refinement, but actually exists in nature, is rendered clear even by the examination of the present question. In fact, no regular relation subsists between the intensity of the cachexia and the number of organs secondarily affected ; cancer of the uterus, which, habitually gives rise to most intense general reaction, rarely induces, as has been stated, extensive secondary formation ; nor is it exceedingly uncommon to find numerous organs affected in subjects who have suffered to a slight amount only from cachectic symptoms. The necessary inference from these facts seems to be that the diathesis and the cachexia are produced in different modes ; and as we have seen that strong probabilities argue in favour of the production of the former by cancerous impregnation of the venous blood, we cannot do otherwise than reject the agency of this in respect of the cachexia. Yet it must be confessed, that until an extended investigation into the condition of the blood at all periods and under all circumstances of the disease shall have been undertaken, these questions are destined to remain undecided."

On the *treatment* of carcinoma, to which Mr. Wells proceeds, we fancy we need say nothing. The interest of such a section is, unfortunately, to come. We cannot, however, conclude without expressing the very favourable opinion we entertain of the manner in which Mr. Wells has executed his task. It reflects credit both on his industry and judgment.

